# Supplementary material for: Competition-cooperation in the chemoautotrophic ecosystem of Movile Cave: first metagenomic approach on sediments
Source: Environ Microbiome. 2022 Aug 17;17:44. doi: 10.1186/s40793-022-00438-w (PMC9386943; doi:10.1186/s40793-022-00438-w)
Supplement: Supplementary file 1 — Additional file 1. Fig. S1 The display of sediment samples collected from Movile Cave. Photographic images of the sediments. [file 40793_2022_438_MOESM1_ESM.docx]

| **Supplementary Figure S1** | | |
| --- | --- | --- |
|  | | |
| 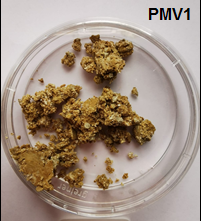 | 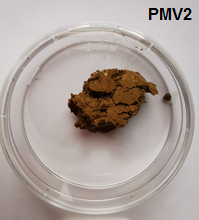 | 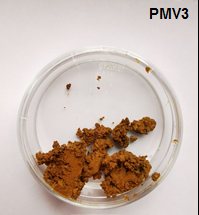 |
| 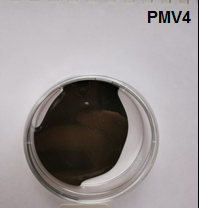 | 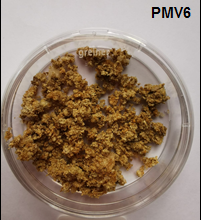 | 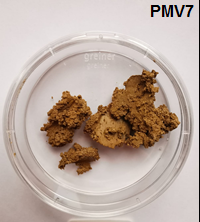 |
| **Supplementary Figure S1.** The display of sediment samples collected from Movile Cave. | | 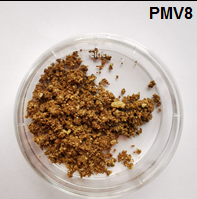 |
