# Supplementary material for: Competition-cooperation in the chemoautotrophic ecosystem of Movile Cave: first metagenomic approach on sediments
Source: Environ Microbiome. 2022 Aug 17;17:44. doi: 10.1186/s40793-022-00438-w (PMC9386943; doi:10.1186/s40793-022-00438-w)
Supplement: Supplementary file 2 — Additional file 2. Table S1 Minimal media composition used in the reconstruction of metaGEMs and community simulations. Minimal compounds (primarily inorganic) that may be in the inorganic environment or can result from microbial metabolic traits (e.g., sulfur and nitrogen oxidation/reduction, CO2 fixation, methanotrophy) previously shown in Movile Cave environment. The compound name and abbreviation are in accordance with the BIGG database (http://bigg.ucsd.edu/ ). Table S2 Metagenome scale metabolic models (metaGEMs) reconstruction and community simulation parameters, results generated and used in this study. Table S3 Codes used for CarveMe v.1.5.1 [41] metabolic model reconstructions (metaGEMs) and SMETANA v.1.1.0 [43] community simulations. [file 40793_2022_438_MOESM2_ESM.docx]

**Supplemental Tables S1-S3**

**Table S1.** Minimal compounds (primarily inorganic) that may be in the inorganic environment or can result from microbial metabolic traits (e.g. sulfur and nitrogen oxidation/reduction, CO2 fixation, methanotrophy) previously shown in Movile Cave environment. The compound name and abbreviation are in accordance with the BIGG database (<http://bigg.ucsd.edu/>).

| Compound | Abbreviation | Compound | Abbreviation |
| --- | --- | --- | --- |
| O_2_ | o2 | chromite | cro2 |
| H_2_O | h2o | chromate | cro4 |
| Ca^2+^ | ca2 | Pb | pb |
| carbonic acid | h2co3 | Pb_2_ | pb2 |
| bicarbonate | hco3 | uranium (IV) | u4 |
| CO | co | uranium (VI) | u6 |
| CO_2_ | co2 | arsenate | aso4 |
| H^+^ | h | arsenite | aso3 |
| hydroxide ion HO | oh1 | tungstate | tungs |
| Cl^-^ | cl | ammonium | nh4 |
| fluoride | f | nitrogen | n2 |
| Co^2+^ | cobalt2 | ammonia | nh3 |
| Cu^2+^ | cu2 | nitric oxide | no |
| iron | fe | nitrate | no3 |
| Fe^2+^ | fe2 | nitrite | no2 |
| Fe^3+^ | fe3 | nitrous oxide | n2o |
| K^+^ | k | hydroxylamine | ham |
| Mg | mg2 | sulfur | s |
| Mn^2+^ | mn2 | hydrogen sulfide | h2s |
| molybdate | mobd | sulfate | so4 |
| Na^+^ | na1 | sulfite | so3 |
| Ni^2+^ | ni2 | thiosulfate | tsul |
| phosphate | pi | thiosulfate | thios |
| Zn^2+^ | zn2 | trithionate | tton |
| cadmium | cd2 | tetrathionate | tet |
| vanadium (V) | v5 | methan | ch4 |
| vanadium (IV) | v4 | methanol | meoh |
| Hg^2+^ | hg2 | acetate | ac |

**Table S2.** Metagenome scale metabolic models (metaGEMs) reconstruction and community simulation parameters and results generated and used in this study.

| **PARAMETERS** | | **RESULTS** | |
| --- | --- | --- | --- |
| **Set of reconstructed GEMs**  **(CarveMe)** | **Community simulation (SMETANA)** | **Global interaction**  **within the *sample community***  **(MRO/MIP, competition/cooperation)** | **Detailed cross-feeding**  **dependency *across conditions***  **(upper/lower galleries, Smetana score = 1)** |
| **no_gap_fill**  (only genetic evidence) | **complete media**  (no environmental constraints) | **Yes** | **Yes** |
|  | **minimal media**  (constrained to a minimal environment) | **NO*** | **NO*** |
| **gap_fill**  (to obtain biomass on a  minimal media) | **complete media**  (no environmental constraints) | **Yes** | **Yes** |
|  | **minimal media**  (constrained to a minimal environment) | **Yes** | **Yes** |

*Note*: ***** Models reconstructed only on genetic evidence cannot reproduce growth on minimal media. Results used in this study.

**Table S3.** Codes used for CarveMe v. 1.5.1 (Machado et al., 2018) metabolic model reconstructions (metaGEMs) and SMETANA 1.1.0 (Zelezniak et al., 2015) community simulations.

|  | | **PARAMETERS** | **CODE** |
| --- | --- | --- | --- |
| **GEMs reconstruction (CarveMe)** | | **no_gap_fill** | $ carve MAG.faa --fbc2 -u (grampus/ gramneg/archaea) -o model.xml |
|  |  | **gap_fill** | $ carve MAG.faa --fbc2 -u (grampus/ gramneg/archaea) --gapfill minimal.media --mediadb my.minimal.media .tsv -o model.xml |
| **MEMOTE** | |  | $ carve MAG.faa --fbc2 -u (grampus/ gramneg/archaea) --gapfill minimal.media --init minimal.media --mediadb my.minimal.media .tsv -o model.xml |
| **Community simulations (SMETANA)** | **Global interaction**  **(MRO/MIP)** | **no_gap_fill**  **complete media** | $ smetana *.xml --flavor bigg --molweight --global --debug |
|  |  | **gap_fill**  **minimal media** | $ smetana *.xml --flavor bigg --molweight -m minimal.media --mediadb my.minimal.media.tsv --global --debug |
| **Community simulations (SMETANA)** | **Detailed cross-feeding**  **(Smetana score)** | **gap_fill**  **complete media** | $ smetana *.xml --flavor bigg --molweight --detailed --debug |
|  |  | **gap_fill**  **minimal media** | $ smetana *.xml --flavor bigg --molweight -m minimal.media --mediadb my.minimal.media.tsv --detailed --debug |
| [**https://carveme.readthedocs.io/en/latest/index.html**](https://carveme.readthedocs.io/en/latest/index.html) | | | |
| [**https://smetana.readthedocs.io/en/latest/index.html**](https://smetana.readthedocs.io/en/latest/index.html) | | | |
