# Supplementary material for: Competition-cooperation in the chemoautotrophic ecosystem of Movile Cave: first metagenomic approach on sediments
Source: Environ Microbiome. 2022 Aug 17;17:44. doi: 10.1186/s40793-022-00438-w (PMC9386943; doi:10.1186/s40793-022-00438-w)
Supplement: Supplementary file 5 — Additional file 5. Fig. S2 The number of reads from the 7 metagenomes mapped back to the reference sequences of the marker genes. Fig. S3 The overall number of reads per metagenome mapped to the reference sequences of all considered marker genes (nitrogen fixation (nifH/D/K), carbon fixation (cbbL/S, aclA/B, acsA(cooS)/B)). Table S10. Reference sequences for the marker genes used in predicting nitrogen and carbon fixation potential in sediments of Movile Cave based on the metagenomic read mapping analysis. [file 40793_2022_438_MOESM5_ESM.docx]

**Supplementary Figure S2-S3**

| 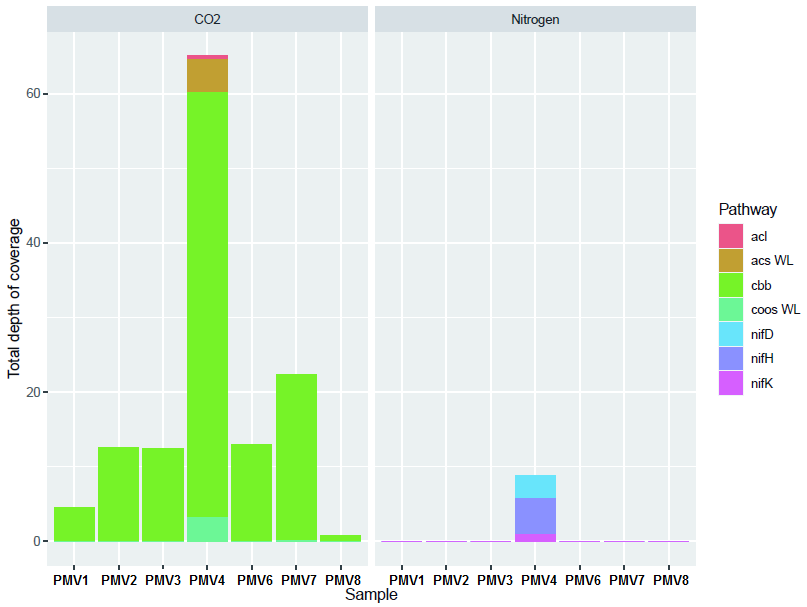 |
| --- |
| **Supplementary Figure S2.** The number of reads (expressed as total depth of coverage**) from the 7 metagenomes mapped back to the reference sequences of the marker genes. The genes reference sequences can be viewed in the Additional file 5: Table S10. |
| ****** On the y axes, the ***total depth of coverage*** is defined as the number of bases mapped to the sequences divided by the sequence's size. |
| 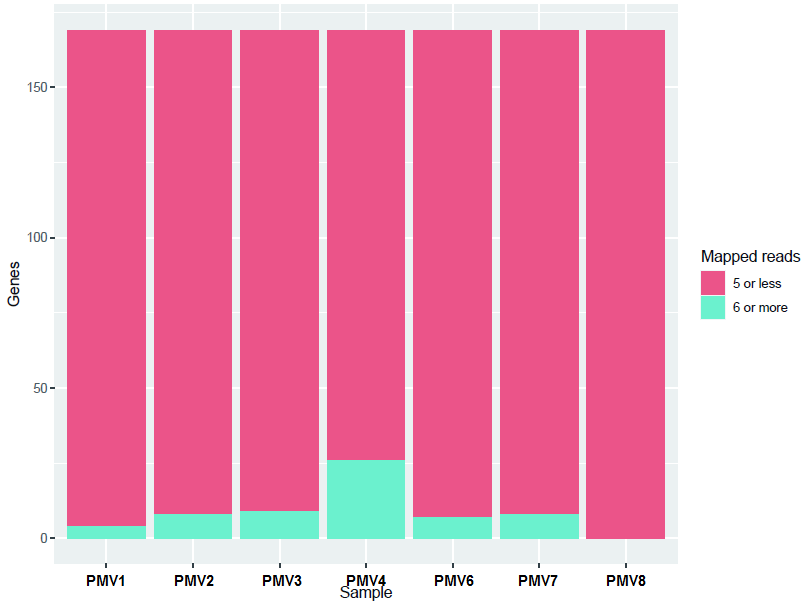 |
| **Supplementary Figure S3.** The overall number of reads per metagenome/sample mapped to the reference sequences of all considered marker genes (nitrogen fixation (*nifH/D/K*), carbon fixation (*cbbL/S*, *aclA/B*, *acsA(cooS)/B*)). |

**Supplementary Tables S10**

**Table S10.** Reference sequences for the marker genes used in predicting nitrogen and carbon fixation potential in sediments of Movile Cave based on the metagenomic read mapping analysis.

| **Pathway** | ***Gene*** | **KEGG Orthology** | **KEGG Organism/CDS entry** |
| --- | --- | --- | --- |
| **Nitrogen metabolism**  **Nitrogen fixation** | ***nifH*** | [K02588](https://www.kegg.jp/entry/ko:K02588) | kpv:KPNIH29_17525 |
|  |  |  | ppol:X809_05005 |
|  |  |  | ppoy:RE92_06765 |
|  |  |  | mmad:MMJJ_01420 |
|  |  |  | mmad:MMJJ_09380 |
|  |  |  | dsd:GD606_12195 |
|  |  |  | dsd:GD606_19425 |
|  |  |  | cbut:ATN24_18575 |
|  |  |  | avn:Avin_49000 |
|  |  |  | avn:Avin_01380 |
|  | ***nifD*** | [K02586](https://www.kegg.jp/entry/ko:K02586) | kpv:KPNIH29_17530 |
|  |  |  | ppol:X809_05010 |
|  |  |  | ppoy:RE92_06760 |
|  |  |  | mmad:MMJJ_01390 |
|  |  |  | dsd:GD606_12210 |
|  |  |  | dsd:GD606_19410 |
|  |  |  | cbut:ATN24_18590 |
|  |  |  | avn:Avin_48990 |
|  |  |  | avn:Avin_01390 |
|  | ***nifK*** | [K02591](https://www.kegg.jp/entry/ko:K02591) | kpv:KPNIH29_17535 |
|  |  |  | ppol:X809_05015 |
|  |  |  | mmad:MMJJ_01380 |
|  |  |  | dsd:GD606_12220 |
|  |  |  | dsd:GD606_19405 |
|  |  |  | cbut:ATN24_18595 |
|  |  |  | avn:Avin_01400 |
|  |  |  |  |
| **Pathway** | ***Gene*** | **KEGG Orthology** | **KEGG Organism/CDS entry** |
| **Carbone metabolism** | ***cbbL/rbcL*** | [K01601](https://www.kegg.jp/entry/ko:K01601) | mox:DAMO_2165 |
| **Carbon fixation** |  |  | mca:MCA2743 |
|  |  |  | metu:GNH96_04105 |
| **Calvin–Benson–Bassham cycle (CBB)** |  |  | mmai:sS8_2433 |
|  |  |  | msl:Msil_1195 |
|  |  |  | mtun:MTUNDRAET4_0602 |
|  |  |  | reh:H16_B1395 |
|  |  |  | afr:AFE_1691 |
|  |  |  | afr:AFE_2155 |
|  |  |  | afr:AFE_3051 |
|  |  |  | paut:Pdca_16240 |
|  | ***cbbS/rbcS*** | [K01602](https://www.kegg.jp/entry/ko:K01602) | mox:DAMO_2166 |
|  |  |  | mca:MCA2744 |
|  |  |  | metu:GNH96_04100 |
|  |  |  | mmai:sS8_2432 K01602 |
|  |  |  | msl:Msil_1196 K01602 |
|  |  |  | mtun:MTUNDRAET4_0601 |
|  |  |  | reh:H16_B1394 |
|  |  |  | afr:AFE_1690 |
|  |  |  | afr:AFE_3052 |
|  |  |  | paut:Pdca_16250 |
| **Reverse tricarboxylic acid cycle (rTCA)** | ***aclA*** | [K15230](https://www.kegg.jp/entry/ko:K15230) | spal:FM071_07485 |
|  |  |  | ssei:FJR45_08955 |
|  |  |  | sku:Sulku_0667 |
|  |  |  | smul:SMUL_0067 |
|  |  |  | sinu:IMZ28_08250 |
|  |  |  | cmed:FE773_04940 |
|  |  |  | cte:CT1088 |
|  |  |  | cpc:Cpar_1009 |
|  |  |  | cph:Cpha266_1306 |
|  |  |  | cli:Clim_1231 |
|  |  |  | sul:SYO3AOP1_1055 |
|  |  |  | tam:Theam_1021 |
|  |  |  | dte:Dester_1134 |
|  | ***aclB*** | [K15231](https://www.kegg.jp/entry/ko:K15231) | spal:FM071_07490 |
|  |  |  | ssei:FJR45_08960 |
|  |  |  | sku:Sulku_0666 |
|  |  |  | smul:SMUL_0066 |
|  |  |  | sinu:IMZ28_08255 |
|  |  |  | cmed:FE773_04935 |
|  |  |  | cte:CT1089 |
|  |  |  | cpc:Cpar_1008 |
|  |  |  | cph:Cpha266_1307 |
|  |  |  | cli:Clim_1232 |
|  |  |  | sul:SYO3AOP1_1056 |
|  |  |  | tam:Theam_1022 |
|  |  |  | dte:Dester_1135 |
| **Wood-Ljungdahl (WL) pathway** | ***acsA(cooS)*** | [K00198](https://www.kegg.jp/entry/ko:K00198) | dbk:DGMP_17830 |
|  |  |  | dbk:DGMP_17900 |
|  |  |  | dal:Dalk_2379 |
|  |  |  | dal:Dalk_0680 |
|  |  |  | dalk:DSCA_26710 |
|  |  |  | dalk:DSCA_27610 |
|  |  |  | dalk:DSCA_28100 |
|  |  |  | dek:DSLASN_16750 |
|  |  |  | dek:DSLASN_29000 |
|  |  |  | dek:DSLASN_47710 |
|  |  |  | dti:Desti_0232 |
|  |  |  | clj:CLJU_c09110 |
|  |  |  | clj:CLJU_c17910 |
|  |  |  | clj:CLJU_c37670 |
|  |  |  | cck:Ccar_07140 |
|  |  |  | cck:Ccar_08590 |
|  |  |  | cck:Ccar_18845 |
|  |  |  | cck:Ccar_23090 |
|  |  |  | pbif:KXZ80_13960 |
|  |  |  | pbif:KXZ80_14960 |
|  |  |  | cdf:CD630_01740 |
|  |  |  | cdf:CD630_07160 |
|  |  |  | roc:HF520_11320 |
|  |  |  | tem:JW646_03340 |
|  |  |  | tem:JW646_10835 |
|  |  |  | tem:JW646_19155 |
|  |  |  | dsy:DSY4442 |
|  |  |  | dsy:DSY4173 |
|  |  |  | dsy:DSY2630 |
|  |  |  | dsy:DSY1653 |
|  |  |  | dhd:Dhaf_3792 |
|  |  |  | dhd:Dhaf_1166 |
|  |  |  | dhd:Dhaf_0882 |
|  |  |  | dhd:Dhaf_2798 |
|  |  |  | dai:Desaci_0709 |
|  |  |  | dai:Desaci_0260 |
|  |  |  | dai:Desaci_2975 |
|  |  |  | drs:DEHRE_00735 |
|  |  |  | aacx:DEACI_2511 |
|  |  |  | aacx:DEACI_0147 |
|  |  |  | aacx:DEACI_1162 |
|  |  |  | awo:Awo_c10740 |
|  |  |  | cthm:CFE_0653 |
|  |  |  | cthm:CFE_1617 |
|  |  |  | cthm:CFE_1666 |
|  |  |  | cthm:CFE_0163 |
|  |  |  | cthm:CFE_0173 |
|  |  |  | cthm:CFE_1919 |
|  |  |  | chy:CHY_0736 |
|  |  |  | chy:CHY_0034 |
|  |  |  | chy:CHY_0085 |
|  |  |  | chy:CHY_1824 |
|  |  |  | mta:Moth_1972 |
|  |  |  | mta:Moth_1203 |
|  |  |  | adg:Adeg_0335 |
|  |  |  | aar:Acear_0983 |
|  |  |  | aar:Acear_1305 |
|  |  |  | toc:Toce_0761 |
|  |  |  | toc:Toce_0335 |
|  |  |  | toc:Toce_0800 sted:SPTER_06840 |
|  |  |  | sted:SPTER_13670 |
|  |  |  | sted:SPTER_01630 |
|  |  |  | bpit:BPIT_02260 |
|  |  |  | jet:L3J17_07160 |
|  |  |  | tpi:TREPR_3069 |
|  |  |  | dtp:JZK55_05490 |
|  | ***acsB*** | [K14138](https://www.kegg.jp/entry/ko:K14138) | dbk:DGMP_17890 |
|  |  |  | dal:Dalk_0681 |
|  |  |  | dalk:DSCA_26720 |
|  |  |  | dek:DSLASN_16760 |
|  |  |  | dti:Desti_0231 |
|  |  |  | clj:CLJU_c37550 |
|  |  |  | cck:Ccar_18785 |
|  |  |  | pbif:KXZ80_13900 |
|  |  |  | cdf:CD630_07280 |
|  |  |  | roc:HF520_11260 |
|  |  |  | tem:JW646_10895 |
|  |  |  | dsy:DSY1652 |
|  |  |  | dhd:Dhaf_2797 |
|  |  |  | dai:Desaci_2974 |
|  |  |  | drs:DEHRE_00705 |
|  |  |  | aacx:DEACI_2512 |
|  |  |  | awo:Awo_c33680 |
|  |  |  | cthm:CFE_1665 |
|  |  |  | chy:CHY_1222 |
|  |  |  | mta:Moth_1202 |
|  |  |  | adg:Adeg_0336 |
|  |  |  | aar:Acear_1304 |
|  |  |  | toc:Toce_0813 |
|  |  |  | sted:SPTER_05160 |
|  |  |  | bpit:BPIT_02250 |
|  |  |  | jet:L3J17_07150 |
|  |  |  | tpi:TREPR_3067 |
|  |  |  | dtp:JZK55_05480 |
